# Tribute of Respect to the Memory of Dr. J. P. Harrison, of Cincinnati, Ohio

**Published:** 1850-02

**Authors:** 


*Tribute of Respect to the Memory of Dr. J. P. Harrison, of Cincinnati, Ohio.*

On the invitation of a number of medical gentlemen, a highly respectable meeting of members of the American Medical Association, belonging to Boston and its vicinity, was convened, for the purpose of paying a tribute of respect to the memory of Dr. John P. Harrison, of Cincinnati, first Vice President of the Association. This meeting was held at the Library of the Boston Society of Natural History, on Tuesday, the 23d of October, and was duly organized by the choice of Dr. Abel L. Pierson, of Salem, as Chairman, and Dr. Henry J. Bowditch, of Boston, as Secretary. The meeting thus opened, was addressed by Dr. J. C. Warren, as follows:

*Mr. Chairman:* The death of our distinguished colleague, Dr. Harrison, which we are at this time assembled to deplore, was announced to us a few weeks since. He fell a victim to the epidemic cholera, which so violently invaded the city of his residence, after a protracted struggle against it, during which he was instrumental in giving relief to many of his fellow-citizens. About the time of the first appearance of this disease, we had a letter from him, in which he spoke in terms showing he had no apprehension in regard to himself.

This gentleman was one of the ablest practitioners in the United States. In the West he was considered as without a superior, had been elevated to the office of Prof. of therapeutics and materia medica, and, in 1845, published a valuable work, in two volumes, on these subjects. He also produced one of the best reports that was made to the American Medical Association, at their meeting in Boston, in the spring of 1849. In theoretical opinion he inclined to solidism. But, while we are not disposed to agree with him on this subject, we must admit that he has defended his position with ability, and in a manner calculated to check the violence of the current now running in favor of humoral pathology.

At the late meeting of the American Medical Association, he was chosen first Vice President of that body, and would, no doubt, have received the general suffrage as President at the next election. These are some of the considerations which entitle his memory to the respectful notice of his professional brethren. He had, also, other claims of a more general nature. While warm and decided in discussion, he was not dogmatical, and gave an agreeable influence to all he said, by the openness and amenity of the manner in which he said it. Another title which he has to the respect of the profession and the eommunity, is derived from the noble manner in which he contended against the fatal epidemic. Most of us here present are of opinion, no doubt, that we, who have enlisted ln the war against disease, must stand to our post, whatever may be the danger, and abide by the resolution to sell our lives as dearly as we can. Some, however, consider it fair, doubtless, that a physician, who has labored through the period of one generation, should excuse himself from any extraordinary efforts. Such was not the opinion of Dr. Harrison, and he continued to expose himself to the disease tili he was destroyed by it.

Called on, as we are, by so many circumstances, to pay some tribute of respect to the virtues of our departed colleague, it will be quite unnecessary, I know, to present any other reasons for doing it, to this meeting. But I cannot pass over another consideration, yet more general in its nature than any I have yet mentioned. Our country consists of many members as yet but imperfectly united in sentiment and policy. Every year that passes over our heads brings with it new materials for cementing us together. One of the best calculated to accomplish this object is a harmony of opinion as to our scientific institutions, and as to the best methods of improving the intellectual character of the nation. In this way a great deal has been done by professional and other associations, extended through the country. Cincinnati, for example, which we formerly considered so remote from us, has, through the intervention of the American Medical Association, become to many of us a neighbor in whose prosperity we feel an interest. The expression of this interest would naturally excite a corresponding feeling on the part of our profession there, and thus serve to invigorate the growth of a patriotic sentiment of regard.

In order to express on our part, the views which this meeting have taken on the subject, which has called them together, I beg leave to propose the following resolutions:

1. *Resolved*, That the members of the American Medical Association, resident in Boston and its vicinity, though personally acquainted with Dr. Harrison for but a short period, have known him sufficiently to estimate the importance of his loss.

2. *Resolved*, That the talents, amenity of manners, and persevering labors of Dr. Harrison, both literary and practical, are worthy of honorable notice from the medical profession.

3. *Resolved*, That the circumstances under which he fell a victim to the fatal epidemic, that has ravaged the country during the past season, entitles his memory to the grateful recollection of all the friends of humanity, and, although we are far removed from the scene of his labors, we join sincerely in the regrets occasioned by his untimely death.

4. *Resolved*, That these proceedings be communicated to his friends in Cincinnati, and the Medical College, of which he was a Professor, and that they be presented for publication in the American Journal of the Medical Sciences, and other Medical Journals.

These resolutions were unanimously adopted, and it was likewise voted that Dr. Warren's remarks should be published with them.

The meeting was then dissolved.

